# Supplementary material for: Inhibiting Myostatin Expression by the Antisense Oligonucleotides Improves Muscle Wasting in a Chronic Kidney Disease Mouse Model
Source: Int J Mol Sci. 2025 Mar 27;26(7):3098. doi: 10.3390/ijms26073098 (PMC11988723; doi:10.3390/ijms26073098)
Supplement: Supplementary file 1 [file ijms-26-03098-s001.zip › ijms-3494389-supplementary.pdf]

Supplementary Figures

A. B.

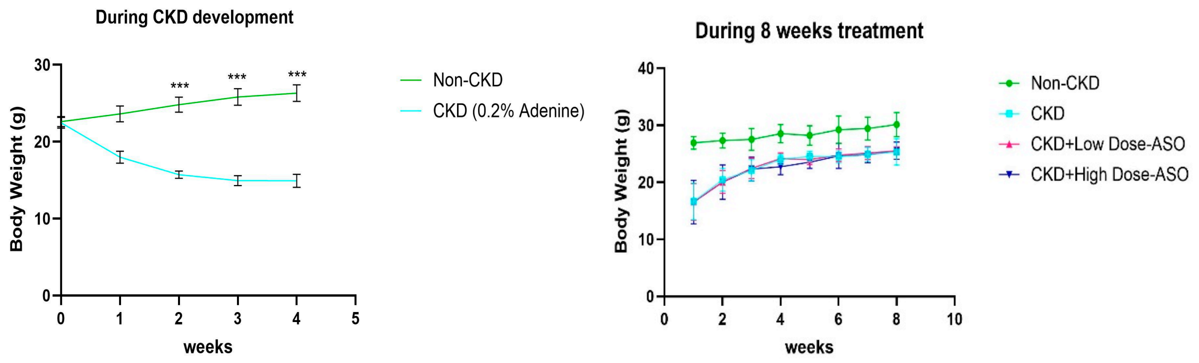

Supplementary Figure S1. Mouse body weight changes. (**A**) The figure compares the body weight changes between non-CKD and CKD mice during a 4-week feeding period with 0.2% adenine and a regular diet. After the development of CKD (when the mice were 12 weeks old), body weight was markedly decreased in the CKD group. The error bars are ±SD; n=8-12. (**B**) The figure compares the body weight changes among non-CKD, CKD, CKD + Low Dose-ASO, and CKD + High Dose-ASO groups. At the end of the experiment, when the mice were 20 weeks old, no significant increase in body weight was observed in the treatment groups (CKD + Low Dose-ASO and CKD + High Dose-ASO) compared to the CKD group. The error bars are ±SD; n=8 per group. Statistical significance was determined by two-way ANOVA followed by Tukey's multiple comparison test. ***p<0.001.

A. **B.**

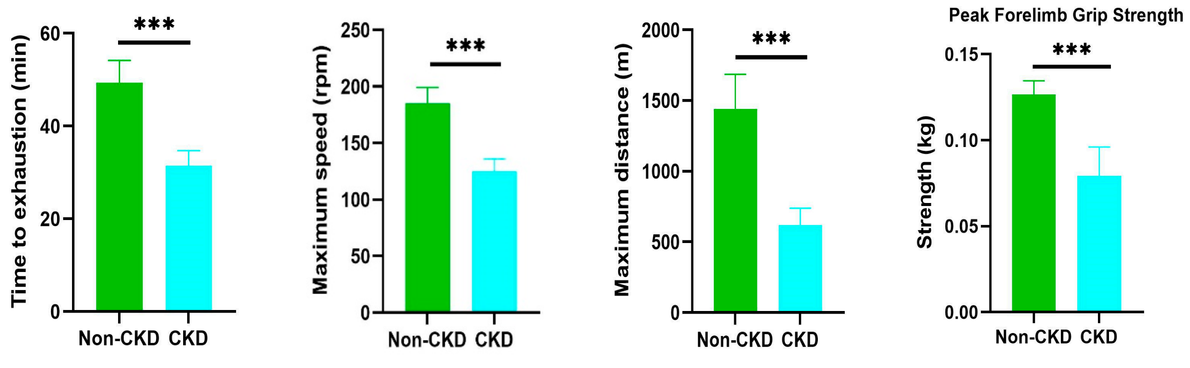

Supplementary Figure S2. Muscle strength and function measurement after CKD development (when mice were 12 weeks old). (**A**) The figure compares the time to exhaustion, maximum speed, and distance traveled by non-CKD and CKD mice on the treadmill after the development of CKD. (**B**) The figure compares the peak forelimb grip strength between non-CKD and CKD mice after feeding a normal diet supplemented with 0.2% adenine for 4 weeks. The error bars are ±SD; n=8-12 per group. The statistical significance was determined using an unpaired t-test. ***p<0.001.

A.

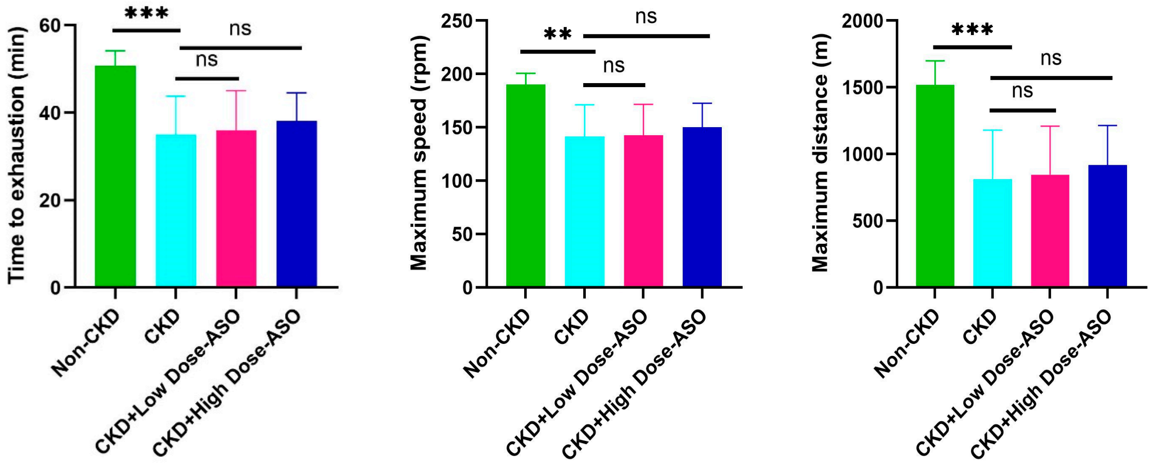

B.

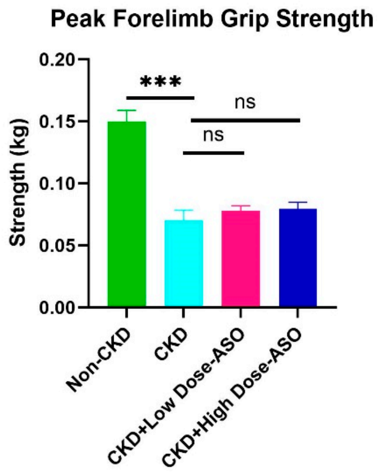

Supplementary Figure S3. Muscle strength and function measurement after 3 weeks of ASO treatment (when the mice were 15 weeks old). (**A**) The figure compares the time to exhaustion, maximum speed, and distance traveled by the non-CKD, CKD, CKD + Low Dose-ASO, and CKD + High Dose-ASO mice on the treadmill after 3 weeks of ASO treatment. (**B**) The figure compares the peak forelimb grip strength among the non-CKD, CKD, CKD + Low Dose-ASO, and CKD + High Dose-ASO groups after 3 weeks of ASO treatment. The Error bars are ± SD; n=8 per group. The statistical significance was determined using one-way ANOVA followed by Tukey’s multiple comparison test. ***$p<0.001$, ns; no significant.

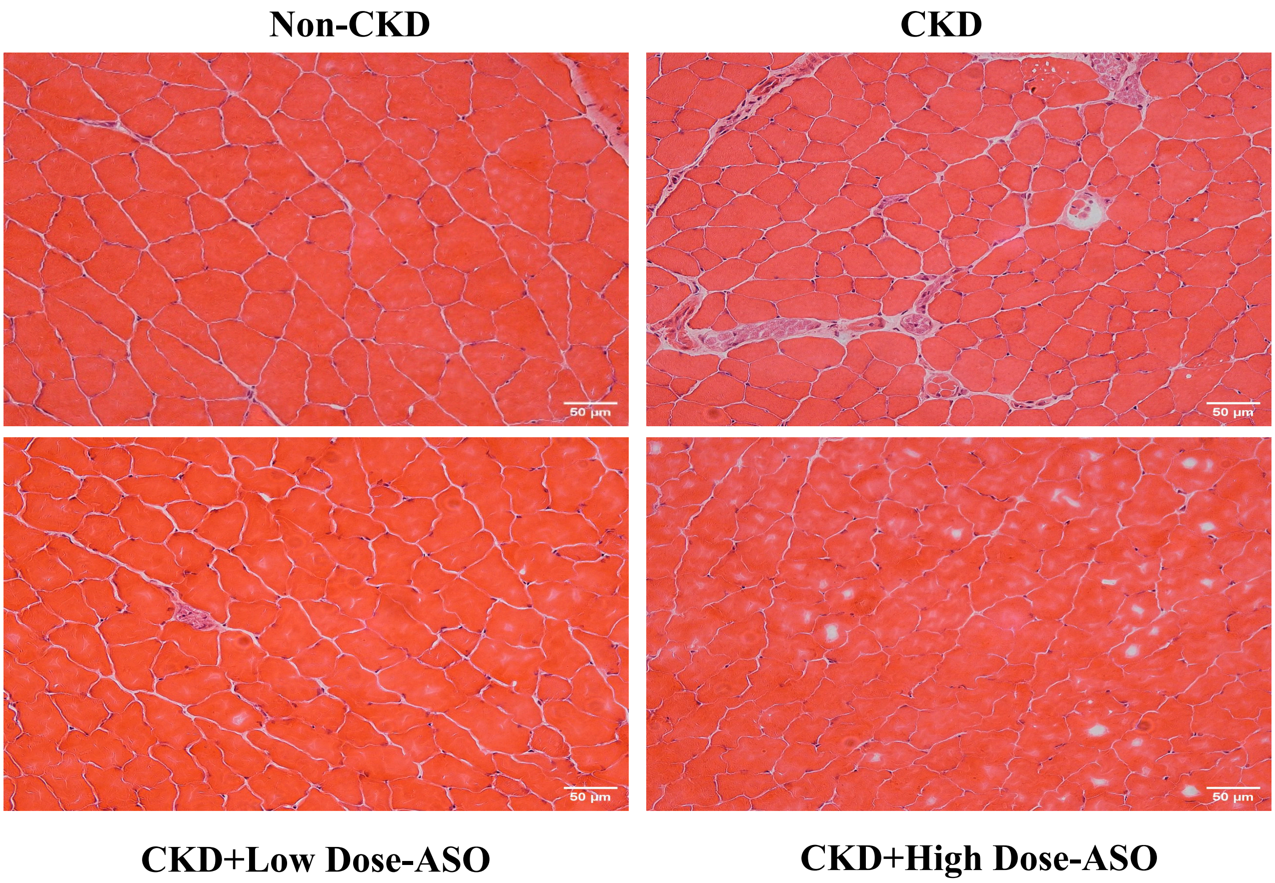

Supplementary Figure S4. Hematoxylin and Eosin staining of muscle. This figure shows the normal histology of the gastrocnemius muscle after HE staining. Scale bar: 50µm (Original magnification 20×)
